# Video-based robotic surgical action recognition and skills assessment on porcine models using deep learning

**DOI:** 10.1007/s00464-024-11486-3

**Published:** 2025-01-13

**Authors:** Nasseh Hashemi, Matias Mose, Lasse R. Østergaard, Flemming Bjerrum, Mostaan Hashemi, Morten B. S. Svendsen, Mikkel L. Friis, Martin G. Tolsgaard, Sten Rasmussen

**Affiliations:** 1 Department of Clinical Medicine, Aalborg University, Aalborg, Denmark; 2 Nordsim – Centre for Skills Training and Simulation, Aalborg University Hospital, Aalborg, Denmark; 3 ROCnord – Robot Centre, Aalborg University Hospital, Aalborg, Denmark; 4 Department of Health Science and Technology, Aalborg University, Aalborg, Denmark; 5 Copenhagen Academy for Medical Education and Simulation, Center for Human Resources and Education, The Capital Region of Denmark, Copenhagen, Denmark; 6 Gastrounit, Surgical Section, Copenhagen University Hospital - Amager and Hvidovre, Hvidovre, Denmark; 7 Department of Clinical Medicine, University of Copenhagen, Copenhagen, Denmark; 8 Department of Computer Science, Aalborg University, Aalborg, Denmark; 9 Department of Urology, Aalborg University Hospital, Aalborg, Denmark

**Keywords:** Robot-assisted surgery, Artificial intelligence, Action regcognition, Skills assessment

## Abstract

**Objectives:**

This study aimed to develop an automated skills assessment tool for surgical trainees using deep learning.

**Background:**

Optimal surgical performance in robot-assisted surgery (RAS) is essential for ensuring good surgical outcomes. This requires effective training of new surgeons, which currently relies on supervision and skill assessment by experienced surgeons. Artificial Intelligence (AI) presents an opportunity to augment existing human-based assessments.

**Methods:**

We used a network architecture consisting of a convolutional neural network combined with a long short-term memory (LSTM) layer to create two networks for the extraction and analysis of spatial and temporal features from video recordings of surgical procedures, facilitating action recognition and skill assessment.

**Results:**

21 participants (16 novices and 5 experienced) performed 16 different intra-abdominal robot-assisted surgical procedures on porcine models. The action recognition network achieved an accuracy of 96.0% in identifying surgical actions. A GradCAM filter was used to enhance the model interpretability. The skill assessment network had an accuracy of 81.3% in classifying novices and experiences. Procedure plots were created to visualize the skill assessment.

**Conclusion:**

Our study demonstrated that AI can be used to automate surgical action recognition and skill assessment. The use of a porcine model enables effective data collection at different levels of surgical performance, which is normally not available in the clinical setting. Future studies need to test how well AI developed within a porcine setting can be used to detect errors and provide feedback and actionable skills assessment in the clinical setting.

**Supplementary Information:**

The online version contains supplementary material available at 10.1007/s00464-024-11486-3.

## Introduction

Surgical performance is directly associated with the intraoperative and postoperative outcome [1–3]. This also applies to robot-assisted surgery (RAS), where insufficient training and inadequate surgical skills can compromise the clinical outcome [3, 4].

A substantial amount of work has been done to create assessment tools for RAS, such as the Global Evaluative Assessment of Robotic Skills (GEARS) score, for use in surgical training and evaluation of surgical performance [5, 6]. However, these assessment tools often depend on an experienced surgeon being present or reviewing video recordings to assess performance [4, 7]. This is resource-demanding, time-consuming, and can be prone to rater bias and interrater variability [3, 4].

Recent emerging technologies based on artificial intelligence (AI) and the subfields of machine learning and deep learning have led to a new field in surgical data science that seeks to assess and evaluate surgical procedures automatically without the need for human assessors [2, 3]. Currently, deep learning models, particularly convolutional neural networks (CNN), combined with other approaches, are among the most common techniques for surgical action recognition [2, 3]. The state-of-the-art in surgical assessment involves the use of a base model, such as CNN or Vision Transformer (ViT), with a temporal aspect, viewing frames as timeseries instead of individual pictures [3, 8, 9]. Unlike the CNN which uses filters to extract features of pictures, the ViT utilizes different patches across the image at the same time [3, 8, 9]. Experimental methods, such as Fusion Key Value (Fusion-KV), integrate multiple data modalities, including video, event data, and kinematic data, alongside various machine learning and deep learning models for more precise results [2, 10]. Although promising, AI solutions generally fail to generalize when presented with different surgical procedures or new datasets from other settings, mainly because of the lack of large and diverse high-quality datasets [3, 8]. To address the pressing need for more robust machine learning models, the present study focused on the development of a deep learning model trained on a highly diverse dataset with multiple procedures and participants with varying surgical skill levels.

We previously presented a method for acquiring and preparing video-based robotic surgical data for machine learning implementation using open-source solutions [4]. Extracting data from video recordings enables access to large amounts of data from different surgical procedures [9]. In this study, we present a method using video-based data to create a deep learning algorithm that can recognize basic surgical actions, such as dissection and suturing, in a diverse dataset with different intra-abdominal procedures performed on in vivo porcine models. Thus, we aimed to further classify surgeons based on short-segment analyses that can be used for immediate feedback in future.

## Methods

### Study setting and participants

The study was conducted at the Biomedical Laboratory of Aalborg University Hospital, Aalborg, Denmark. We collected data from different RAS procedures using in vivo porcine models. All procedures on porcine models were approved by The Danish Animal Experiments Inspectorate under the Danish Veterinary and Food Administration (ID: 2018-15-0201-01392).

The Robot Center Nord (ROCNord) at Aalborg University Hospital conducts several RAS courses annually, and participants were included if they participated in one of these RAS courses. Based on prior work, we defined two groups of participants: experienced (> 100 RAS procedures) and novice (< 100 RAS procedures) [2, 3, 8, 11, 12]. The study was approved by The North Denmark Region Committee on Health Research Ethics (ID: 2021-000438) and the Regional Research Review Board under the Danish Data Protection Agency (ID: 2021-246).

All video footage and labels were anonymized and are available at our GitHub repository www.Github.com/NasHas [13] for open-source use in future research. Furthermore, all Python scripts created and used in this study can also be found at our GitHub repository for open-source use [13].

### Data capture and extraction

The method of collecting and preparing data was based on our previously published study on the acquisition and usage of robotic surgical data for machine learning algorithms [4]. We only utilized step one and step four of our previous method and thus did not extract event data or movement data from the surgeons in the current study [4]. The da Vinci Surgical System (DVSS) surgeon console was connected to two HDMI to USB Video Capture Cards (VCCS) from Ozvavzk (one for each ocular output of the surgical robot).

Video footage was recorded using OBS Studio (Open Broadcaster Software Studio, Wizards of OBS, OBS Studio v. 27.2.4, 64-bit) with a recording set at 15 frames per second (FPS) and 2560 × 720 pixels to record both ocular outputs of the surgical robot (some initial videos were recorded at other resolutions and different FPS; please refer to our previous study on data collection [4]). The videos were cropped into separate right and left 1280 × 720 (or 1920 × 1080 [4]) views, and sequences where nothing of relevance occurred (such as cleaning of camera and change of instruments) were cut out using Free Video Crop, RZSoft Technology Co. Ltd, v. 1.08, and the command-line software FFmpeg.

### Preparation of robotic surgical data

In this study, we used temporal labeling, in which each surgical event was marked by a timestamp at its beginning and end. We marked two primary label categories representing the basic elements and actions of surgery; ‘suturing’ and ‘dissection’ [1, 2, 4, 7, 14, 15]. The suturing label covers a suture action from the initial suture positioning in the instrument to the final knot tying [2, 7, 16]. We used two labels to indicate the type of suture (single or running) and two labels for suture actions (needle driving and suture handling, including positioning and knot tying). The dissection labels were divided into three subcategories; general dissection (blunt, sharp, or combined techniques such as two-handed dissection and coagulation with cutting), clip application (applying clips before cutting, hot or cold), and hemostasis control (locating and stopping bleeding) [1, 2]. Only general dissection was analyzed, while the other subcategories were excluded. All actions were labeled from their initiation to their conclusion.

The labels were created manually using the Behavioral Observation Research Interactive Software (BORIS, v. 7.13.8) [17], and all labels were created by NH.

### Pre-processing

The video footage was sampled at 1 FPS using our script in Python 3.8. To accommodate the temporal aspect of the network, we stacked five consecutive frames into sequences (all frames in one sequence had the same label; see Supplementary Fig. 1). This was also performed using Python script. We found that there were three main groups of sequences: sequences where either suturing or dissection occurred and sequences where both suturing and dissection occurred. Using the lowest denominator, we chose to omit the last category because of the low number of frames and ensured that the number of sequences in the remaining two groups was balanced as much as possible to avoid overrepresentation of one class. The information was saved as a CSV file.

For the skills assessment, each video had a label revealing whether the video was conducted by a novice or experienced participant and was split into sequences of 10 s.

The raw footage also contains information bars in the lower and upper parts of the picture, and these parts of each frame were cropped, and consecutively, all frames were resized from their original resolution (either 1280 × 720 or 1920 × 1080) to 256 × 256 pixels to lower the computational requirements.

After creating image sequences, cropping, and resizing, the complete dataset was split into three groups: training, validation, and test sets to perform the final evaluation of the best model. We chose a split of ∼ 80% of the data for the training set, ∼ 10% for the validation set, and ∼ 10% for the test set. We also ensured that videos of each participant would only be used exclusively in one specific dataset, that is, no participants would be present in the two datasets at the same time. Split and balancing were performed automatically based on the total number of sequences using our available Python script. Therefore, minor differences in the number of sequences between the suturing and dissection groups could occur (see Supplementary Tables 1 and 2). For skills assessment, we balanced the datasets manually so that procedures performed by both novice and experienced groups were included in the training set, and only procedure types used in the training set were used in the validation and test sets, see Supplementary Table 2.

We then combined the training and validation sets (~ 90% of the data) and used K-fold cross-validation to estimate the performance of our model. K-fold cross-validation splits the dataset into K-folds, in this case five folds, and leaves onefold for testing, while training on the remaining folds. It then iterates through this process until all the folds have been trained and tested. Performance accuracies are saved for each fold, and the mean performance and standard deviation of the cross-validation are presented (see Supplementary Table 3), which provides a more generalized estimate of how the model would perform on unseen data. We used K-fold cross-validation to fine-tune the network parameters before finally using the best parameters to train a new model on the full dataset (the training and validation set) and on the unseen test set.

### Architecture of the neural network

**Fig. 1:**
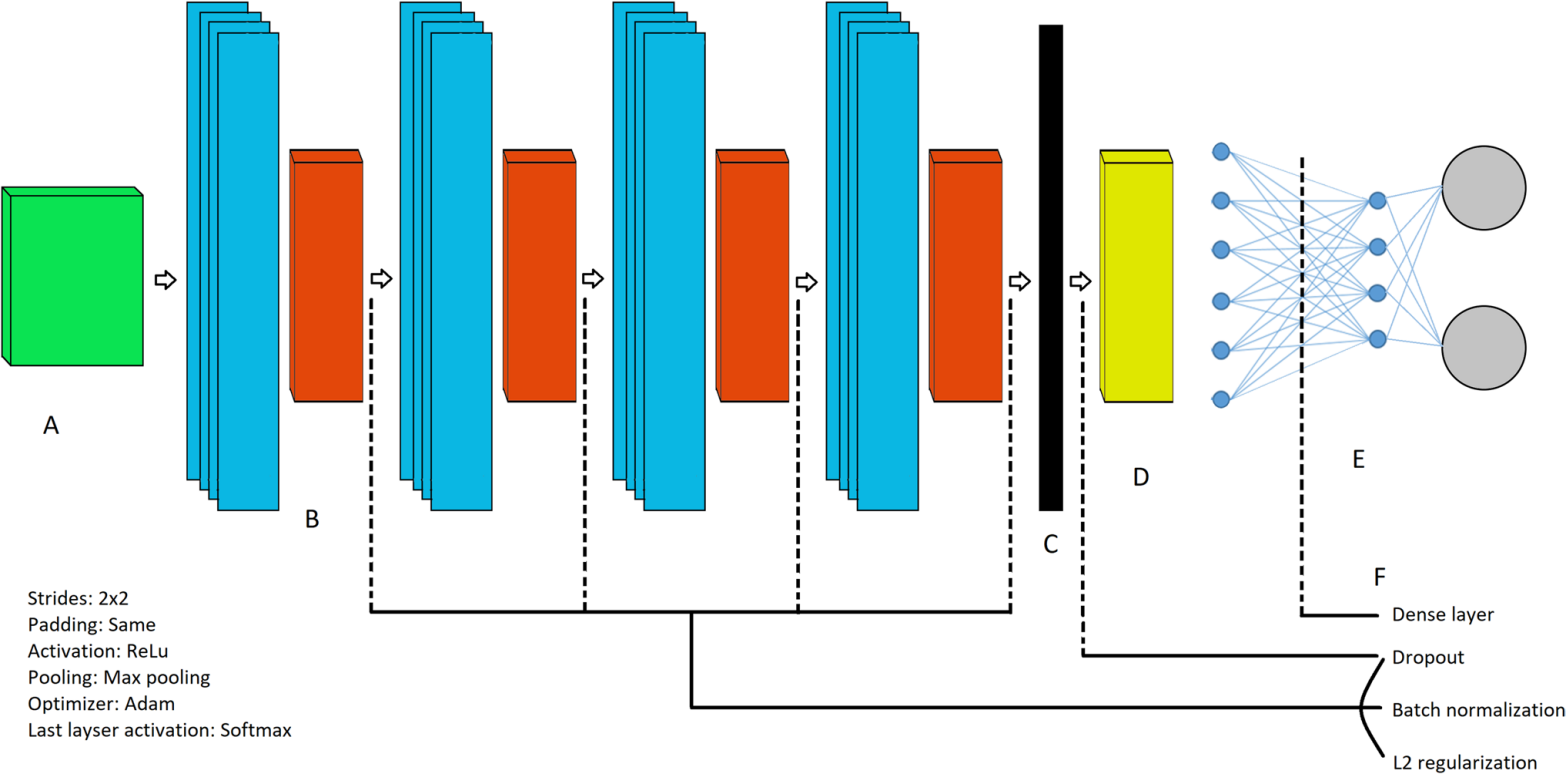
The final architecture of the CNN LSTM networks were the same for both primary action classification and for skill assessment. It consists of an initial input layer (**A**) followed by four convolutional layers each accompanied by a pooling layer (**B**). Then the inputs are flattened (**C**) before being inputted in the LSTM layer (**D**) which then outputted to a fully connected dense layer through a Softmax activation function that in the end provides two probabilities (**E**), one for each class (Suturing vs. Dissection and Novice vs. Experienced). For skills assessment we used the same architecture but added an extra dense layer (64 neurons) and used random dropout 0.20, batch normalization and L2 regularization rate 0.01, to overcome overfitting and compensate for lack of data (**F**)

Our network combines a CNN to extract spatial features with a Long Short-Term Memory (LSTM) layer incorporation of temporal information, see Fig. 1. For a more detailed explanation of the network structure, see Supplementary Text 1.

We split the dataset into small batches of eight five-second sequences and ran up to 50 training cycles (epochs), using early stopping to avoid overfitting.

We developed two versions of the network: one for classifying actions (suturing vs. dissection) and another for assessing skill level (novice vs. experienced). The skills assessment was limited by having only four videos available for testing, so we applied techniques like dropout, batch normalization, regularization, and one extra dense layer before the last layer to prevent overfitting.

### Model evaluation

We evaluated our models by generating confusion matrices and calculating the accuracy, recall/sensitivity, precision, F1-score, true-positive rates, and false-positive rates.

To increase interpretability, we calculated the predictive certainty of the action recognition network by performing predictions and obtaining probabilities for each class, and then saving the highest probability of each prediction and comparing them with the ground true labels. This was then plotted visually along with the maximum, mean, and minimum probabilities. The probability distribution can be viewed as an expression of the certainty behind the decision to classify a certain sequence as a certain class.

For the skills assessment network, we used the true- and false-positive rates to plot the ROC curve and AUC for the entire test set as well as for the subsets.

We also used Gradient-weighted Class Activation Mapping (Grad CAM) to produce a localization map that highlights regions of importance in the image sequences analyzed by the algorithm. For skill assessment, we plotted all predictions during a complete video to determine the sections in which misclassifications occurred.

## Results

We included data from 21 surgeons who participated in the study (16 novices and 5 experienced RAS surgeons; Table 1). In total, 16 different intra-abdominal RAS procedures were performed on in vivo porcine models (from either urological, general surgical, or gynecological courses; Table 2) and our open-source dataset [13]. From the 130 recorded procedural videos, the temporal annotations of suturing and dissection were turned into sequences of 5 s and 10 s for the training, validation, and testing of the neural networks (see Supplementary Tables 1 and 2).

**Table 1:** Demographics of the participants

	Novices	Experienced
Number of participants, no	16	5
Sex (Men/Women), no	8/8	4/1
Age (mean (SD)), years	40.1 (7.1)	47.4 (9.8)
Handedness (L/R), no	4/12	2/3
Occupation & specialty		
Resident/Registrar	10	
Specialist doctor	6	5
Urology	11	4
Gynecology/Obstetrics	2	
Thoracic surgery Gastrointestinal surgery	1 2	1
Robotic surgical experience (total cases)		
< 100	16	
> 100		5

*L/R* Left/Right, *SD* standard deviation

**Table 2:** Overview of procedures and recordings

	Number of participants	16	5
Number	Procedure	Novices	Experienced
1	Salpingectomy (fallopian)^a^	13	2
2	Bladder defect suturing^b^	21	3
3	Lymph node dissection^c^	24	8
4	Ureter dissection^d^	16	
5	Ureter implantation^e^	7	
6	Ureter anastomosis	2	
7	Nephrectomy^f^	5	1
8	Partial nephrectomy^g^	9	2
9	Bowel puncture^h^	4	3
10	Cystectomy		1
11	Pyeloplasty^i^	2	
12	Rectum dissection^j,k^	3	
13	Hysterectomy^k^	1	
14	Mesh in rectum^k^	2	
15	Bowel anastomosis	1	1
16	Release of vessels	1	
	Sum of recordings	109	21
	Total recordings	130	

a Was done either with vascular clip of uterine artery or with burning

b Some procedures were done with initial bladder incision, others were done with pre-incised holes in the bladder

c The lymph node dissection was either done on rectal nodes or at other locations in the pelvis (pelvic nodes)

d Differences in location for dissection could occur

Some participants begins the dissection more cranial, others more caudal

e Was either done with continuous suturing or by way of Taguchi method

f Differences in the beginning and end of the procedure could occur

g The procedure was done with or without dissection (of renal fascia), with or without two layers of suture, and if not with two layers of suture, with either V-lock continuous suture or as single sutures with clips

h Some procedures were done with initial incision, others were done with pre-incised holes in the bowels

i Two recordings were made, one containing the initial cutting and preparation of the ureter and the second with the anastomosis

j One procedure was done alone

Two were done with Hysterectomy and Mesh in rectum as part of a bigger rectopexy procedure

k These were done as part of a bigger rectopexy procedure

K-fold cross-validation in five splits resulted in a mean accuracy of 90% for action recognition and 72% for skill assessment. The full results are from the last round of hyperparameter tuning and are presented in Supplementary Table 3.

### Primary action category network

The CNN LSTM network for primary action classification, classifying suturing and dissection, reached the lowest average validation loss in the 8rd epoch, and the last model of this epoch was used for the final testing.

The model was used to predict the test set, which was then randomly selected and prepared.

The accuracy of the model was 96.0%. This provided an average recall of 96.0%, precision of 96.0%, and F1-score of 96.0% for the model, as shown in Table 3.

**Table 3:** The average recall, precision, and F1-score for the primary action category predictions of the CNN LSTM network

	Recall	Precision	F1-score
Dissection	0.949	0.970	0.959
Suturing	0.970	0.950	0.960
Weighted avg	0.960	0.960	0.960

Finally, the predictive certainty had a mean value of 98.82%, with a maximum probability value of 100% and a minimum probability value of 50.19%. A plot of predictive data is shown in Supplementary Fig. 2.

### Gradient-weighted class activation mapping

**Fig. 2:**
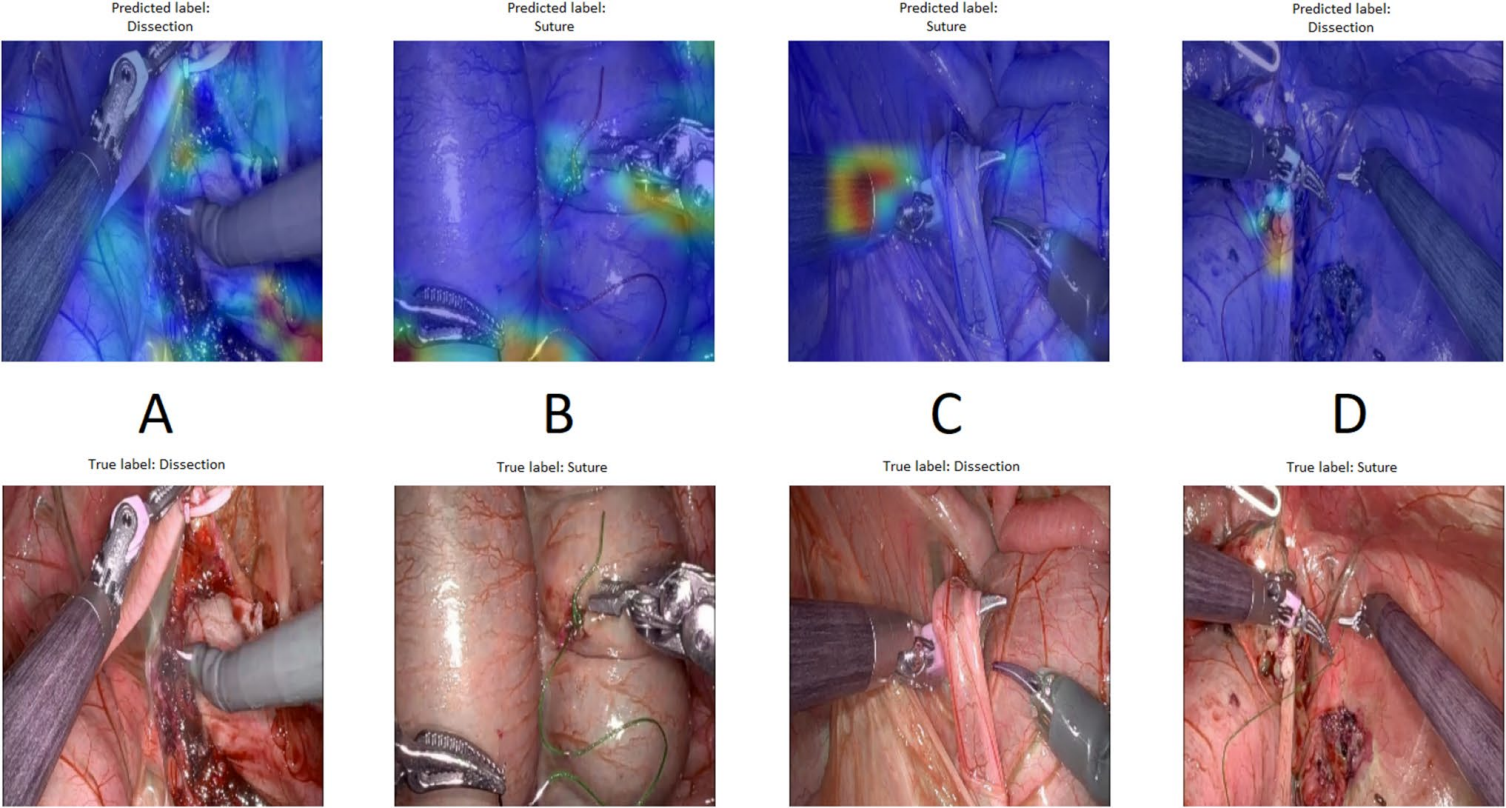
The regions of interest for the algorithm during surgical procedures. **A** and **B** The areas in focus when classifying dissection and suturing, **C** shows a misclassification of a dissection sequence, and **D** shows a misclassification of suturing

To produce a visual explanation of the spatial regions featured by the CNN LSTM network, we used a GradCAM filter. As shown in Fig. 2, examples from the network are illustrated for each category (suturing vs. dissection).

We can see that the network focuses on the tissue and instrument tips during dissection and on the needle, suture, and instrument tips during suturing.

### Skills assessment network

For the skill assessment, the training reached the lowest average loss at the 20th epoch. The final model of this epoch was saved for final testing.

When assessed on the test set, it showed an accuracy of 81.3%, as listed in Table 4.

**Table 4:** The average recall, precision, and F1-score for skills assessment predictions of the CNN LSTM network

	Recall	Precision	F1-score
Experienced	0.943	0.759	0.841
Novice	0.672	0.915	0.775
Weighted avg	0.813	0.833	0.809

This resulted in an average recall of 81.3%, a precision of 83.3%, and an F1-score of 80.9%, as shown in Table 4.

**Fig. 3:**
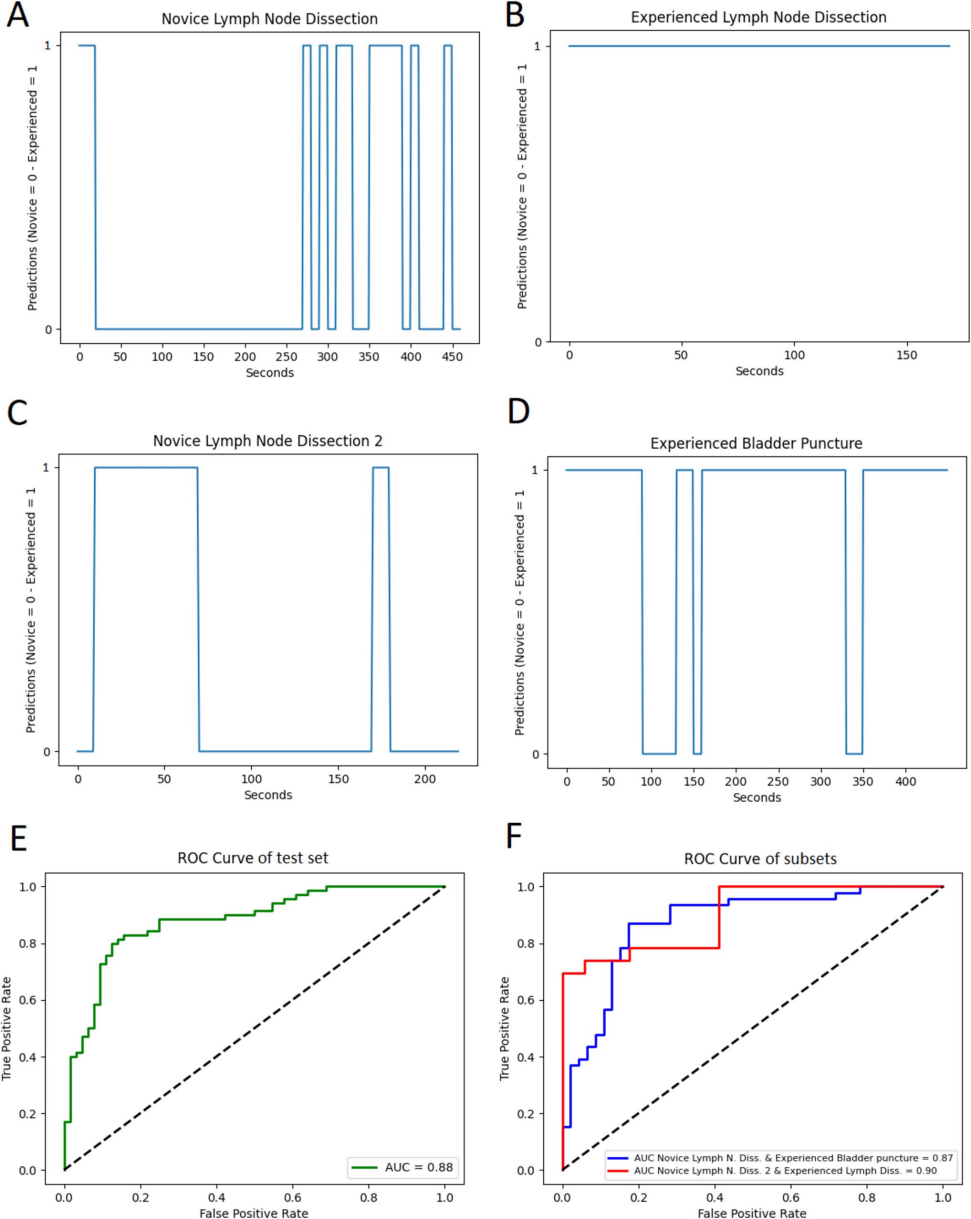
Procedure plots depicting the predictions (novice 0, experienced 1) during the complete procedures of the test set. As can be seen, there were sections of the procedures, where the novice participant was classified as experienced (**A** and **C**), and sections during the procedure ‘Bladder puncture’ where the experienced was classified as a novice (**D**). **B** The experienced was classified as an experienced during the complete procedure. **E**, **F** ROC curves and AUC for the complete test set (**E**) and for the individual subsets (**F**)

The predictions can be plotted for the complete procedure video, providing insight into which parts of the procedure novices were misclassified as experienced and vice versa (see Fig. 3).

Figure 3 shows procedure plots for all test videos and the ROC curves with respective AUC for the complete test set and subset predictions. The ROC curve for the complete test set shows an AUC of 0.88. Comparing the subsets shows an AUC of 0.87 between novice lymph node dissection and experienced bladder puncture and an AUC of 0.90 when comparing novice to experienced surgeons in lymph node dissection.

## Discussion

In this study, we used a novel method [4] to acquire data and train a deep learning network with two sets of configurations for the classification of surgical actions and skills assessment. Both networks demonstrated good performance in a surgically diverse dataset. We used the same network architecture (CNN-LSTM) for both problems, demonstrating the flexibility of the model.

Interest in machine learning-based action recognition or surgical skill assessment of RAS has been increasingly investigated in recent years [2, 3, 18]. Most studies use accuracy to evaluate model performance, as it measures the ratio of correct predictions to total predictions, unlike precision, which focuses specifically on the accuracy of positive predictions [2, 3, 9, 19, 20] or image-level binary classification tasks, such as in this study, the Metrics Reloaded framework supports using accuracy as a primary metric [21]. In addition to accuracy, we also include precision, recall, and F1-score for a more comprehensive evaluation of the results. Regarding action recognition, prior studies have achieved accuracies ranging from 68 to 90% [2, 3, 8]. Similarly, in the context of skill assessment, accuracies range from 76 to 100% when only video data are used [2, 3, 8]. Among state-of-the-art for surgical video assessment is SAIS, which leverages a pre-trained ViT model with a temporal component to identify surgical gestures and evaluate surgical skills [9]. This approach was tested across three hospitals and two surgical procedures (robot-assisted nephrectomy and radical prostatectomy), achieving AUC values exceeding 0.90 for gesture recognition and over 0.80 for both skills assessment and cross-procedure gesture recognition [8]. However, SAIS primarily evaluated on experienced surgeons and also had a more detailed discrimination of surgical gestures [8]. Another notable study utilized a temporal segment network for surgical assessment, combining a CNN with temporal aspect, like our network, to achieve 95% accuracy [10]. This research was conducted using the JIGSAWS dataset for training and testing [9].

Most prior studies have tested their models on the JIGSAWS dataset, which is a public dataset of video and kinematic data made in a highly standardized, controlled dry lab environment [22]. The main limitation of using a small dataset in a controlled environment is overfitting, which occurs when an algorithm is not generalizable to new data from other environments or procedures [2, 3, 22]. The small size of the JIGSAWS dataset makes it difficult to allocate each participant exclusively to training, validation, or test sets, as we have done in the current study, due to the limited number of experts in the JIGSAWS dataset (only two). This raises questions regarding the results of prior studies in this field, as many studies do not explicitly address how they avoid leakage from training to validation and test data [2, 3].

Another problem when training and developing machine learning models based on dry lab data is that they do not generalize to clinical settings. However, data acquisition from clinical settings is difficult and expensive [23]. More importantly, it may be impossible to collect data for training machine learning models that include examples of poor or erroneous performance in the clinical setting, which are needed to train a good model to assess different levels of clinical skills. In recent years, public datasets like CholecTriplet, HeiChole, SAR-RARP50, ESAD, and PSI-AVA have emerged, alongside non-public datasets, such as SAIS and Theator, which is a surgical video database platform [3, 8, 24–28]. These datasets all use endoscopic footage, similar to our dataset. However, CholecTriplet and HeiChole are specific to human laparoscopic cholecystectomy, excluding robotic surgery, while SAR-RARP50, ESAD, PSI-AVA, and SAIS datasets focus on human robot-assisted radical prostatectomy (RARP) procedures [3, 8, 24–28]. Also, procedures were done by experienced surgeons, creating a small variance in both procedures and group of participants [3, 8, 24–28]. Annotation methods also varied, with SAR-RARP50 using only visual annotations, boundary boxes, while the other datasets include both visual and temporal annotations, such as instrument segmentation and time labels [3, 8, 24–28]. We used an in vivo porcine wet-lab setting to allow for the collection of data from multiple procedures across a large variety of surgeons with different skill levels, both novice and experienced [29]. This enabled us to develop a model that was indifferent to the 16 different surgical procedures on which it was trained. We also chose to use temporal annotations of the videos, where each surgical action was defined in the time they occurred, because it represents the most basic and simple way of annotating surgical procedures, especially when aiming to collect greater datasets and streamlining the data processing [30, 31]. Other methods such as spatial annotation using boundary boxes or segmenting instruments or anatomic structures are usually technically harder and require more defined criteria [2, 31]. We chose the two main categories of tasks; suturing and dissection and left out subcategories to avoid unbalancing the classes and because of the skewed frequency the subcategories were used throughout the surgical procedures [4]. We also left the ‘Other’ category, which was described in our previous study as a category for tasks, such as suction and holding [4]. We excluded the “Other” group because it overlapped with the “Suturing” and “Dissection” classes, creating a multilabel issue with skewed balance and technical complexity in this proof-of-concept study. Secondly, the “Other” class was inconsistent, containing varied actions that sometimes resembled “Suturing” or “Dissection” due to shared elements. Thus, the “Other” class was excluded from training and testing. Although previous research has identified subcategories within suturing and dissection, a widely accepted classification system and consensus has not yet been established [1, 2, 7, 16]. Therefore, we adopted broader definitions of suturing and dissection that encompass finer subcategories [1, 2, 7, 16]. Both the suturing and dissection labels were annotated as segments of time using timestamps, which is a function of BORIS [7, 16]. We based our subcategories on previous research which defines various subcategories of both suturing and dissection [1, 2, 7, 16]. For example, our subcategory of general dissection label included sharp dissection (which have previously been defined as spread, hook, push, and peel with any instrument), sharp dissection (hot and cold cut, burn and cut), and combinations (multiple peels either blunt or sharp and dissection with both instruments) [1]. Because of the size of our dataset, a subcategorization of the surgical actions would lead to non-generalizable results [2, 8]. The use of generic tasks is supported by the SAGES framework for annotation of surgical videos [32]. All labels were annotated by a medical doctor who is a clinical trainee in urology. Our use of temporal annotations aligns with the SAGES framework; however, because we did not annotate surgical phases or steps, defining relationships between different parts of the procedures is challenging [32]. Additionally, we did not label segments were nothing of surgical relevance happened (such as cleaning of camera and change of instruments); instead, we removed it during pre-processing, which aligns with the reason of why they should be labeled according to the SAGES framework [32]. We suggest that developing machine learning models in a wet-lab setting will allow easier generalization to the clinical setting, potentially using much smaller amounts of data for transfer learning, as demonstrated in other areas of data science [33–37]. This will be the subject of future research.

Skill assessment annotations were based on a binary classification (experienced vs. novice), defined by operative volume alone. Each procedure was labeled as either ‘experienced’ or ‘novice.’ While this quantitative approach is common, it may not accurately reflect individual technical skill levels, it lacks flexibility, and studies have shown considerable variability in determining the skill levels [33, 34]. We chose a value of 100 cases to distinguish between novice and experienced surgeons. Still, previous studies have used a wide range, from 30 to over 1,000 cases, with thresholds differing by procedure type and medical specialty [2, 3, 8, 11, 12, 33, 38]. A way of generating more flexibility could be to use more degrees of experience and actual ratings of clinical performance. However, our proof-of-concept model demonstrates continuous evaluation on shorter segments during the procedures, unlike assessments such as GEARS, which only gives endpoint evaluation. Continuous evaluation provides surgeons and trainees with identifiable segments of less surgical quality during a procedure, allowing for targeted improvement in future procedures [8, 33]. Another limitation in the skills assessment tasks was our access to experienced robotic surgeons. Future research could benefit from a multicenter approach to gathering more data, addressing the challenge of having few experienced robotic surgeons at a single center [3, 8].

When models fail to deliver results accuracy despite the best possible set of features, there are two main avenues for improvement: using techniques to prevent overfitting and underfitting or gathering more data [39]. A clear limitation of the current study was the number of experienced participants and the overall size of the dataset. The dataset was reduced to comply with the criteria of participant exclusivity in the training, validation, and testing sets and also to balance both novice and experienced groups. Because of the limited skills assessment data, we used different machine learning techniques such as dropout, regularization, batch normalization, and an extra dense layer to make the network more robust and avoid overfitting. All machine learning models, including those used in surgical video analysis, are inherently limited by the quality of the datasets on which they are trained [31]. Biases introduced during the collection of training data can result in models that are less generalizable [31]. A pitfall is the use of datasets with limited variability, which fail to account for differences in surgical approaches, differences in anatomy, or even institutional practices [31]. However, because of the black-box nature of deep learning algorithms, we cannot be sure of which features truly influence a models predictions [40]. The lack of explainability and interpretability has been one of the reasons hindering its implementation [40]. GradCAM has been described as a way to make increase the interpretability of deep learning algorithms, especially CNN [40]. As shown in Fig. 2, the visual representation provides an interpretation of the decisions leading to the algorithm’s choices. Figure 2 shows the individual frames from longer sequences that are input to the LSTM layer. In addition, GradCAM has other limitations, such as problems with localizing multiple occurrences in an image, possible loss of signal because of the up- and down-sampling processes, and problems with the gradients of deep layers of a neural network [40]. It is important to note that our model analyzes not only the spatial features highlighted by GradCAM but also the temporal changes in these regions using the LSTM layer, making decisions based on the entire sequence rather than isolated frames. The use of the LSTM layer allows the model to recognize sequences and patterns over time, which is crucial for distinguishing similar actions with different outcomes [41]. Features that increase both interpretability and explainability are important for gaining the trust of clinicians and helping with the implementation of AI in clinical settings [8, 40, 42]. Future research could focus on methods that incorporate transparency as part of the network architecture or include multiple features simultaneously to increase both interpretability and explainability [40]. Moreover, studies are needed to determine how real-time machine learning feedback impacts the surgical workflow, surgeon attention, performance, and long-term learning.

## Conclusion

Our study demonstrated that machine learning can be used to automate surgical action recognition and skill assessment. The use of in vivo porcine models enables effective data collection at different levels of surgical performance, which is normally not available in the clinical setting. Future studies are needed to test how well machine learning models developed within a porcine setting can be used to detect errors and provide feedback and actionable skills assessment in the clinical setting.

## Supplementary Information

Below is the link to the electronic supplementary material.

Supplementary file1 (DOCX 13 KB)

Supplementary file2 (PNG 849 KB)

Supplementary file3 (DOCX 13 KB)

Supplementary file4 (PNG 43 KB)

Supplementary file5 (PNG 19 KB)

Supplementary file6 (PNG 15 KB)

Supplementary file7 (DOCX 15 KB)

Supplementary file8 (DOCX 14 KB)

Supplementary file9 (DOCX 14 KB)

Supplementary file10 (DOCX 20 KB)
