# Supplementary material for: Video-based robotic surgical action recognition and skills assessment on porcine models using deep learning
Source: Surg Endosc. 2025 Jan 13;39(3):1709–19. doi: 10.1007/s00464-024-11486-3 (PMC11870904; doi:10.1007/s00464-024-11486-3)
Supplement: Supplementary file 1 — Supplementary file1 (DOCX 13 KB) [file 464_2024_11486_MOESM1_ESM.docx]

Supplementary Figure 1:

A schematic of the workflow. A) Data preprocessing combining video frames and labels into labeled sequences and afterward balancing and splitting the sequences into different datasets. B) The training and validation datasets were cross validated to tune hyperparameters. The best parameters were then in a new model which was used to train on the full dataset, including the unseen test-set. C) The final result is either action recognition or skills assessment.
