# Supplementary figures and images for: Video-based robotic surgical action recognition and skills assessment on porcine models using deep learning
Source: Surg Endosc. 2025 Jan 13;39(3):1709–19. doi: 10.1007/s00464-024-11486-3 (PMC11870904; doi:10.1007/s00464-024-11486-3)

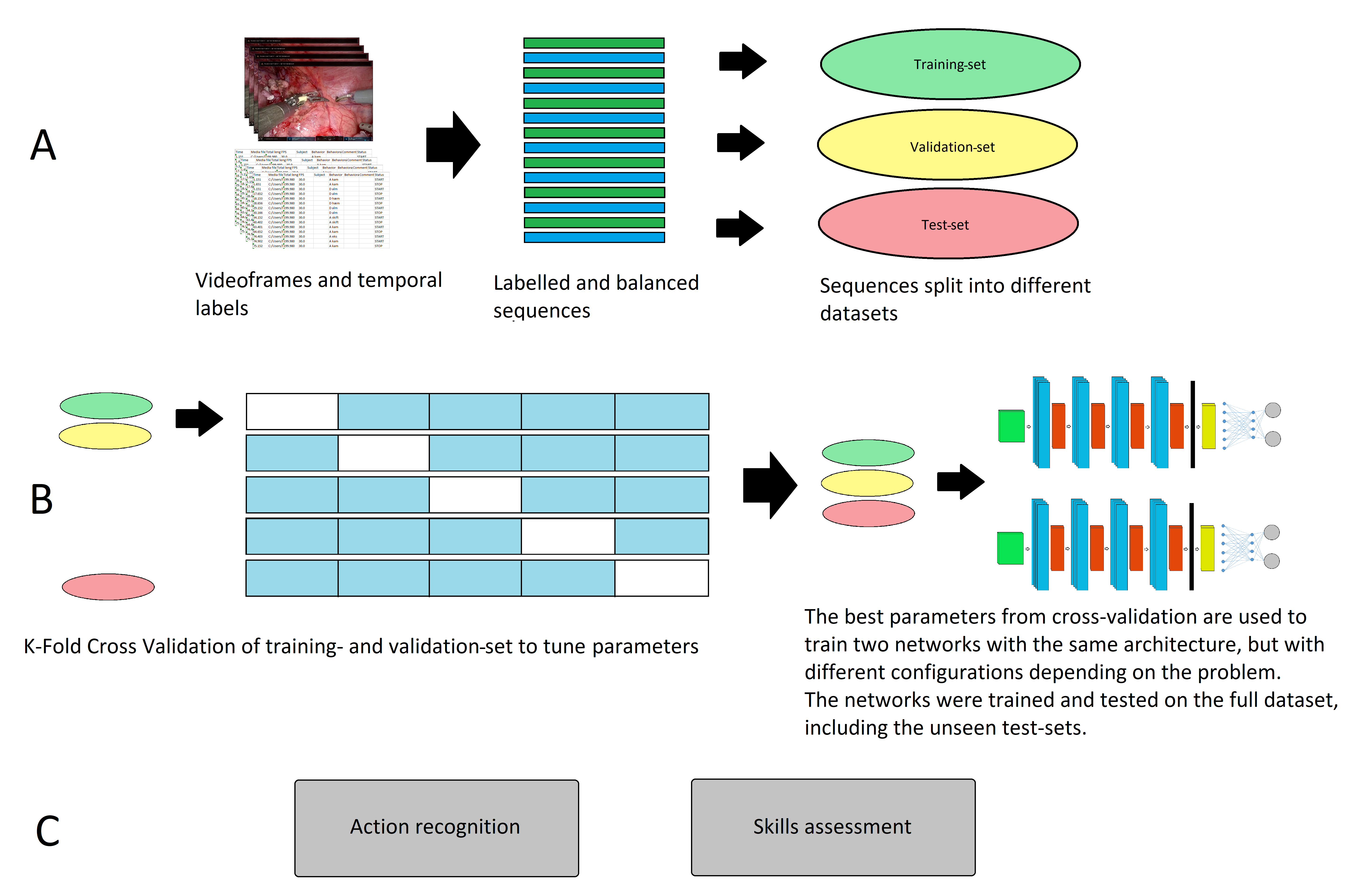

Supplement: Supplementary file 2 — Supplementary file2 (PNG 849 KB) [file 464_2024_11486_MOESM2_ESM.png]

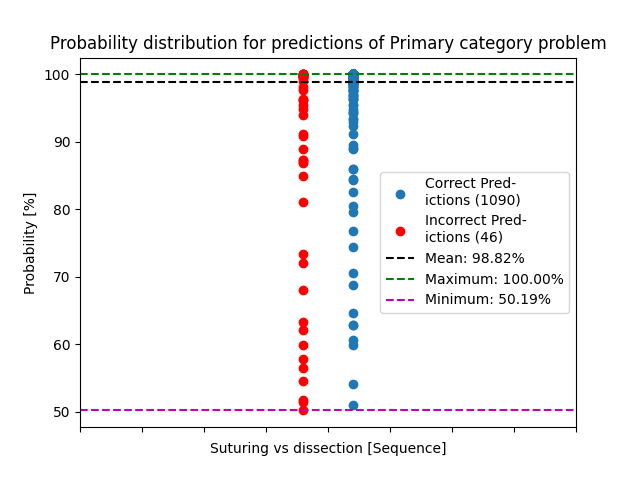

Supplement: Supplementary file 4 — Supplementary file4 (PNG 43 KB) [file 464_2024_11486_MOESM4_ESM.png]

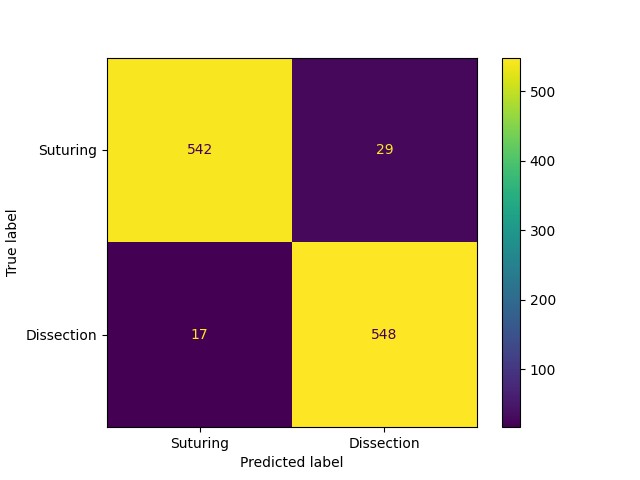

Supplement: Supplementary file 5 — Supplementary file5 (PNG 19 KB) [file 464_2024_11486_MOESM5_ESM.png]

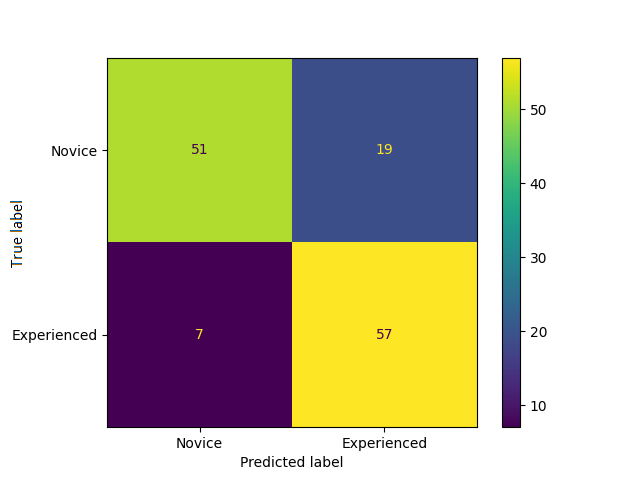

Supplement: Supplementary file 6 — Supplementary file6 (PNG 15 KB) [file 464_2024_11486_MOESM6_ESM.png]
