# Supplementary material for: Video-based robotic surgical action recognition and skills assessment on porcine models using deep learning
Source: Surg Endosc. 2025 Jan 13;39(3):1709–19. doi: 10.1007/s00464-024-11486-3 (PMC11870904; doi:10.1007/s00464-024-11486-3)
Supplement: Supplementary file 3 — Supplementary file3 (DOCX 13 KB) [file 464_2024_11486_MOESM3_ESM.docx]

Supplementary Figure 2:

The figure depicts the 1136 predictions made for the primary action categories by the CNN LSTM network. The blue dots represent the correct predictions, and the red dots represent the incorrect predictions. The maximum, minimum and mean probabilities are also depicted.
