# Supplementary material for: Video-based robotic surgical action recognition and skills assessment on porcine models using deep learning
Source: Surg Endosc. 2025 Jan 13;39(3):1709–19. doi: 10.1007/s00464-024-11486-3 (PMC11870904; doi:10.1007/s00464-024-11486-3)
Supplement: Supplementary file 7 — Supplementary file7 (DOCX 15 KB) [file 464_2024_11486_MOESM7_ESM.docx]

|  | Number of videos used (participants) | Number of frames used | Class Split | Split percentage |
| --- | --- | --- | --- | --- |
| Training | 57 | 49023 | Dissection: 23776 - Suture: 25247 | Dissection: 48.5% - Suture: 51.5% |
| Validation | 9 | 5337 | Dissection: 2538 - Suture: 2799 | Dissection: 47.6% - Suture: 52.3% |
| Test | 10 | 5683 | Dissection: 2858 - Suture: 2825 | Dissection: 50.3% - Suture: 49.7% |

Supplementary Table 1 The videos, frames and balanced data-split used to train, validate, and test the primary action recognition network. The videos used in the datasets can be seen in our repository on Github.
