# Supplementary material for: Video-based robotic surgical action recognition and skills assessment on porcine models using deep learning
Source: Surg Endosc. 2025 Jan 13;39(3):1709–19. doi: 10.1007/s00464-024-11486-3 (PMC11870904; doi:10.1007/s00464-024-11486-3)
Supplement: Supplementary file 8 — Supplementary file8 (DOCX 14 KB) [file 464_2024_11486_MOESM8_ESM.docx]

|  | Number of videos used | Number of frames used | Class Split | Split percentage |
| --- | --- | --- | --- | --- |
| Training | 23 | 10030 | Experienced:4920 - Novice: 5110 | Experienced: 49.1% - Novice: 50.9% |
| Validation | 4 | 1220 | Experienced: 560 - Novice: 660 | Experienced: 45.9% - Novice: 54.1% |
| Test | 4 | 1340 | Experienced: 640 - Novice: 700 | Experienced: 47.8% - Novice: 52.2% |

Supplementary Table 2 The videos, frames and balanced data-split used to train, validate and test the skills assessment network. The videos used in the datasets can be seen in our repository on Github.
