# Supplementary material for: Video-based robotic surgical action recognition and skills assessment on porcine models using deep learning
Source: Surg Endosc. 2025 Jan 13;39(3):1709–19. doi: 10.1007/s00464-024-11486-3 (PMC11870904; doi:10.1007/s00464-024-11486-3)
Supplement: Supplementary file 9 — Supplementary file9 (DOCX 14 KB) [file 464_2024_11486_MOESM9_ESM.docx]

| Fold number | Accuracy (Action recognition) | Accuracy (Skills assessment) |
| --- | --- | --- |
| 1 | 0.92 | 0.53 |
| 2 | 0.84 | 0.36 |
| 3 | 0.89 | 0.99 |
| 4 | 0.92 | 1.00 |
| 5 | 0.91 | 0.70 |
| Mean (SD) | 0.90 (0.03) | 0.72 (0.28) |

Supplementary table 3 Accuracies for the final round of hyperparameter tuning for both networks (action recognition and skills assessment). Mean accuracies and standard deviation are also tabulated.
