# Supplementary material for: Video-based robotic surgical action recognition and skills assessment on porcine models using deep learning
Source: Surg Endosc. 2025 Jan 13;39(3):1709–19. doi: 10.1007/s00464-024-11486-3 (PMC11870904; doi:10.1007/s00464-024-11486-3)
Supplement: Supplementary file 10 — Supplementary file10 (DOCX 20 KB) [file 464_2024_11486_MOESM10_ESM.docx]

*Supplementary Text 1:*

*Architecture of the neural network*

The internal architecture of our network is comprised of a convolutional neural network (CNN) for the extraction of spatial features and a Long Short-Term Memory (LSTM) layer for the incorporation of temporal information, as shown in Figure 1. This provides the ability to extract features and remember information across images and how related features develop over time [17]. To make the training feasible, we split the dataset into smaller batches of eight sequences, each representing five seconds, trained before updating the network parameters. When all data had been through the network, an epoch was reached, and we chose to train for a maximum of 50 epochs, but with an early stop function (which was activated if no improvement was seen in five consecutive epochs).

We developed one network (CNN and LSTM) but used different configurations depending on the problem, one to classify the primary categories of action (suturing and dissection), and one network to assess the skill level (novice or experienced) (see Figure 1).

For the skills assessment, the amount of training, validation, and test videos was sparse and resulted in only four videos being used for testing after the initial balancing of data. Because of the small amount of data, we chose to add dropout (rate 0.02), batch normalization, and L2 regularization (rate 0.01) to further prevent overfitting and to deploy one extra dense layer before the last layer, besides using K-fold cross validation for tuning of parameters, as mentioned earlier.

Neural Network Architecture

Our neural network architecture consists of convolutions, activation functions, pooling, LSTM, and dense layers for effective feature extraction and classification (Figure 1).

Convolutions: Four convolutional layers (64-128-256-512 filters) extract essential features by emplyoing 3×3 filters with 2×2 strides and 'Same' padding.

Activation Function: After convolution, Rectified Linear Unit (ReLU) activation is applied to enhance the filter correlations and retain relevant values [18].

Pooling: Following each convolutional layer, a Max Pooling layer (3 × 3 size, 2 × 2 strides) increases generalizability and reduces computational demands.

Long Short-Term Memory (LSTM): After the convolutional and pooling layers, a flattening operation precedes an LSTM layer with 64 neurons for stable information storage [19].

Last Layer Activation: After the LSTM layer, the final layer is a dense layer, which is a fully connected layer that utilizes the softmax activation function, providing class probabilities. For each problem, we used two output neurons: dissection vs. suturing and novice vs. experienced neurons.

In the skills assessment network, an extra dense layer with 64 neurons preceded the final layer.

Loss Function and Optimizer: We employed the cross-entropy loss function (CELF), which guides the adjustment of weights and biases during training [20]. The Adam Optimizer was employed for efficient parameter updates to enhance network performance.
